# Looking at reality versus watching screens: Media professionalization effects on the spontaneous eyeblink rate

**DOI:** 10.1371/journal.pone.0176030

**Published:** 2017-05-03

**Authors:** Celia Andreu-Sánchez, Miguel Ángel Martín-Pascual, Agnès Gruart, José María Delgado-García

**Affiliations:** 1 Neuro-Com Research Group, Universitat Autònoma de Barcelona, Edifici I, Facultat Ciències de la Comunicació, Campus Bellaterra, Cerdanyola del Vallès, Barcelona, Spain; 2 Division of Neurosciences, Pablo de Olavide University, Seville, Spain; Universidad de Salamanca, SPAIN

## Abstract

This article explores whether there are differences in visual perception of narrative between theatrical performances and screens, and whether media professionalization affects visual perception. We created a live theatrical stimulus and three audio-visual stimuli (each one with a different video editing style) having the same narrative, and displayed them randomly to participants (20 media professionals and 20 non-media professionals). For media professionals, watching movies on screens evoked a significantly lower spontaneous blink rate (SBR) than looking at theatrical performances. Media professionals presented a substantially lower SBR than non-media professionals when watching screens, and more surprisingly, also when seeing reality. According to our results, media professionals pay higher attention to both screens and the real world than do non-media professionals.

## Introduction

Due to the recent expanded digitalization of our world, we are surrounded by screens. In the US, for example, adults spend an average of 8 hours and 47 minutes per day watching screens—with tablets, smart phones, PCs, multimedia devices, video game consoles, DVDs, time-shifted TV, and live TV [1, 2]. Despite media works being composed of an apparent continuity of fixed pictures, on many occasions they seem real to us. However, we can distinguish between reality and drama on the screen, so no one calls the police when seeing a murder in a movie [3]. We know that perception of any narrative (live or on-screen) is divided into segments of attention processed independently by the viewer [4–6]. This segmentation of autonomous narrative elements can be done with some freedom during the perception of the real world. However, when we are watching an audio-visual work, we are also giving attention to segmentations made by the director through edition [7]. It is, then, to be expected that we perceive reality and screens differently. A way to investigate those visual differences is through the quantification of the respective spontaneous blink rates (SBR). Indeed, the emergence of personal computers, giving rise to long periods of intensive work with screens, has a high incidence in SBR: people inhibit their eyeblink rate between 50% and 60% when watching the screen of a computer [8]. Computer Vision Syndrome, linked to screen use, has been well characterized through vision problems, visual accommodation problems, blurred vision, fatigue, and dry eyes [9–12]. Likewise, it is well known that eyeblink rate is affected by performing tasks that require attention. Wong and colleagues, for instance, noted that the rate in ophthalmologic surgeons decreased during the execution of surgery, from 16 to 4 eyeblinks per minute [13]. The connection between eyeblink and attention has been proven in various circumstances [14] including the use of screens [15, 16], or for diagnosis in Autism Spectrum Disorders [17–20]. In addition, a decrease in eyeblink rate has been linked to symptom worsening associated to dry keratoconjunctivitis or dry eye syndrome [8, 21]. It is well known that the narrative in media works can also affect eyeblink frequency and that SBRs synchronize among spectators watching the same movie [22, 23], contributing to the idea that blinking has not only a physiological function [23–25] but also a psychological one [26]. In fact, the importance of eyeblinks in the communicative process has been proven [19, 27], being understood as an attentional marker linked to the hypothetical Default Mode Network (DMN), which would be active when there is no attentional focus in media works [28]. However, there is controversy about the meaning of DMN [29, 30] and how it would be analyzed in media environments. Eyeblinks are also synchronized between listener(s) and talker in a conversation [27], in particular in the pauses and at the end of the discourse. On the other hand, we know that professionalization is a variable that in previous studies has been shown to change audio-visual perception and to provoke perceptive differences, and even structural brain changes, in—for example—musicians [31], athletes [32], or drivers [33, 34]. In this investigation, we had two specific aims: i) to look for differences in SBR evoked by attending the same narrative across a live theatrical performance or across a movie; and ii) to find out whether there are visual perceptive differences in SBR linked to media professionalization.

## Materials and methods

### Subjects

Forty healthy adults (20 non-media professionals and 20 media professionals) with normal or corrected-to-normal visual acuity participated in this study. The average age of the group of non-media professionals (15 men and 5 women) was 43.25 years (*SD* = ± 8.59) and the average time in their professions was 19.4 years (*SD* = ± 10.21). In the group of media professionals (16 men and 4 women), the average age was 44.15 years (*SD* = ± 7.15) and the time in their media professions was an average of 20.2 years (*SD* = ± 8.63). To be included in the media group, it was required that subjects needed to use video edition and to take decisions related to media editing in their everyday work. Thus, media professionals taking part in this experiment were producers, assistant producers, cameramen, image controllers, documentalists, graphic designers, post-production editors, sports commentators, and video editors. Non-media professionals were chosen outside of this criterion. The study had the approval of the Ethics Commission for Research with Animals and Humans (CEEAH) of the University Autònoma de Barcelona, Spain, with reference number 2003. All experiments were performed in accordance with relevant guidelines and regulations. Written informed consent was obtained from all participants.

### Stimuli

We created four stimuli with the same narrative, action, character, and duration: three were movies with different video editing styles, and one was a live play, as shown in S1 Video. Stimulus 1 was a one-shot movie with a static open shot and no cuts; stimulus 2 was a Hollywood-editing-style movie, following classical continuity editing rules, with smooth transitions and perceptive continuity [35]; stimulus 3 was an MTV-editing-style movie, also known as post-classical or video-clip style, characterized by short moved shots, sudden moves in the frame, and a continuous motion of the camera [36, 37]; and stimulus 4 was a live performance that was played out in each experimental session. We used the three most common movie-editing styles for the screened stimuli to be in concordance with the habitual consumption of audio-visual narrative on screens. The duration of stimuli 1, 2, and 3 was 198 s each; the duration of stimulus 4 was adjusted to last approximately 198 s for each subject. The action and narrative of the four stimuli were exactly the same: a man entered, sat, juggled with three balls, worked with a computer and books, ate an apple, looked directly at the spectator/viewer, and left.

### Stimulus presentation

**Fig 1:**
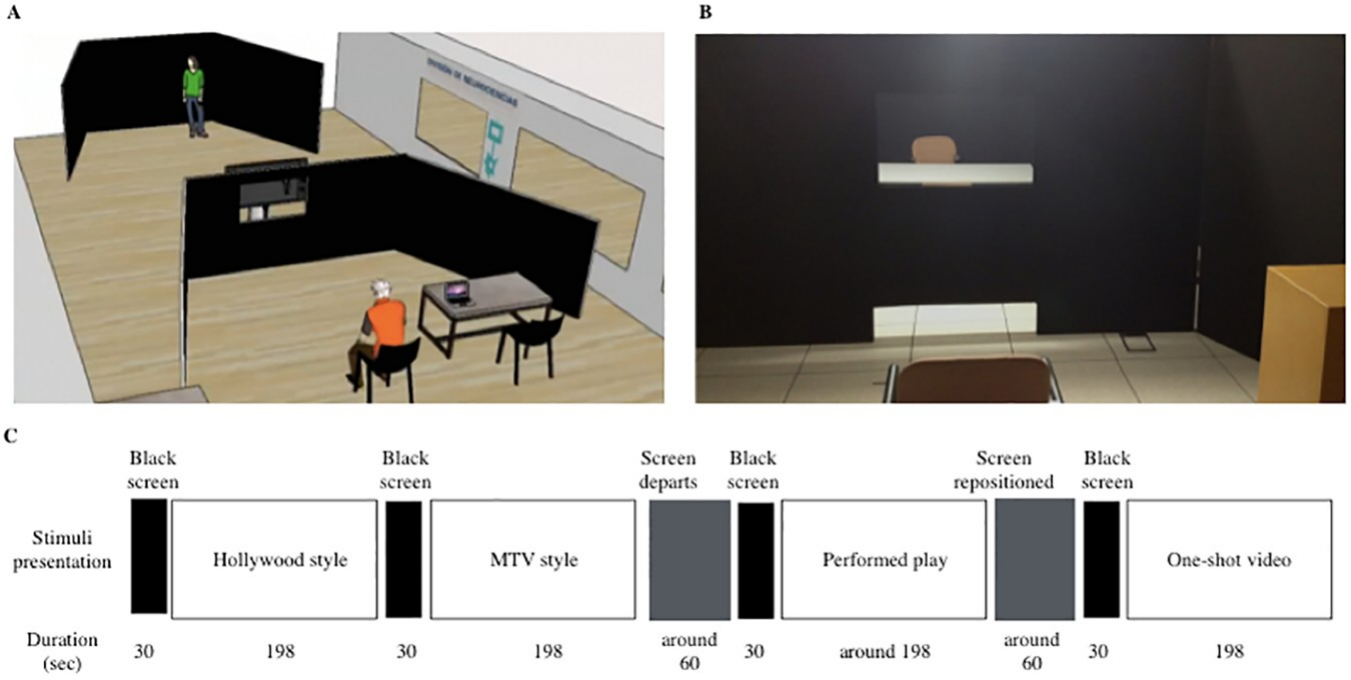
Stimulus presentation. (A) We designed a special stage for carrying out stimulus presentation. It comprised two different areas: at the back, there was a black backdrop with a table where the actor would make the live play presentation; in the front part, there was a black panel with a hole for the screen showing the videos. The screen was placed in the hole for video stimuli and removed for the live play stimulus. (B) The subjects were placed in front of the hole so that they could perceive the play with a black framework. This experimental set-up was aimed at making live and video stimuli experiences as similar as possible. (C) The order of stimulus presentation was randomized over the 24 possible combinations—for example, Hollywood-style movie–MTV-style movie–Performed play–One-shot movie. Each stimulus was preceded by a black screen lasting for 30 s. Whenever the live play had to be presented, the screen used for the videos was removed. It was replaced in the designed hole for the presentation of each video stimulus. The duration of the live play was approximately 198 s, while the duration of each video stimulus was 198 s.

For the presentation of the four stimuli, two black structures were created to define a flexible space: one black structure (4x2m) as a backdrop for the live play, and a second black structure with the same dimensions and a hole in the middle. This hole was designed to fit a screen (Panasonic TH-42PZ70EA, Panasonic Corporation) for the presentation of the video stimuli at a distance of 150 cm from participants (Fig 1A and 1B). For the representation of the live play, the screen was removed and subjects had visual access to the performance, as shown in S1 Video. Paradigm Stimulus Presentation (Perception Research System) was used for stimulus presentation and synchronization of recording. A pause of 30 s preceded each stimulus. During the pause, subjects had a rest period, received no experimental stimulus, and were not monitored. The presentation of the stimuli was randomized using a tetrahedron. Twenty-one of 24 possible combinations were present in the study (Fig 1C).

### Data acquisition and analysis

The EMG activity of the orbicularis oculi muscle was acquired with a wireless system (Enobio®, Neuroelectrics). Silver chloride (Ag/AgCl) electrodes were placed according to the 10–20 system. Electrooculogram, Fp1, and Fp2 channels were carefully placed to detect eyeblinks and other eyelid movements. The Cz channel was used as reference channel during the recording. The subject’s eyes were also visually recorded with an HD video camera (Sony HDR-GW55VE, Sony Corporation). The video camera framed the subject’s face in a close-up.

Eyeblinks were analyzed using two different methods. First, we detected them in the EMG signal with Brainstorm [38] running on MATLAB (The MathWorks Inc.). We filtered the signal from 0.5 Hz to 3 Hz, applied Brainstorm’s eyeblink detector, and carefully checked the results, as proposed by Tadel et al. [39]. Then, and in accordance with Nakano and Kitazawa [27], we checked the results manually with HD-video recordings of the subject’s ocular behavior. Statistical analysis was performed offline using Sigmaplot 11.0 (Systat Software Inc.). We analyzed data following a two-way repeated-measures analysis of variance (ANOVA) and further post hoc analysis using the Holm-Sidak method, with the whole datasets. Data were analyzed for normality and equal variance with the Shapiro-Wilk test. All three video stimuli were analyzed together under the “screen” condition. To avoid inaccuracies in data analysis derived from different duration in the live play stimulus, in each condition we worked with the mean of SBR per minute.

## Results

**Fig 2:**
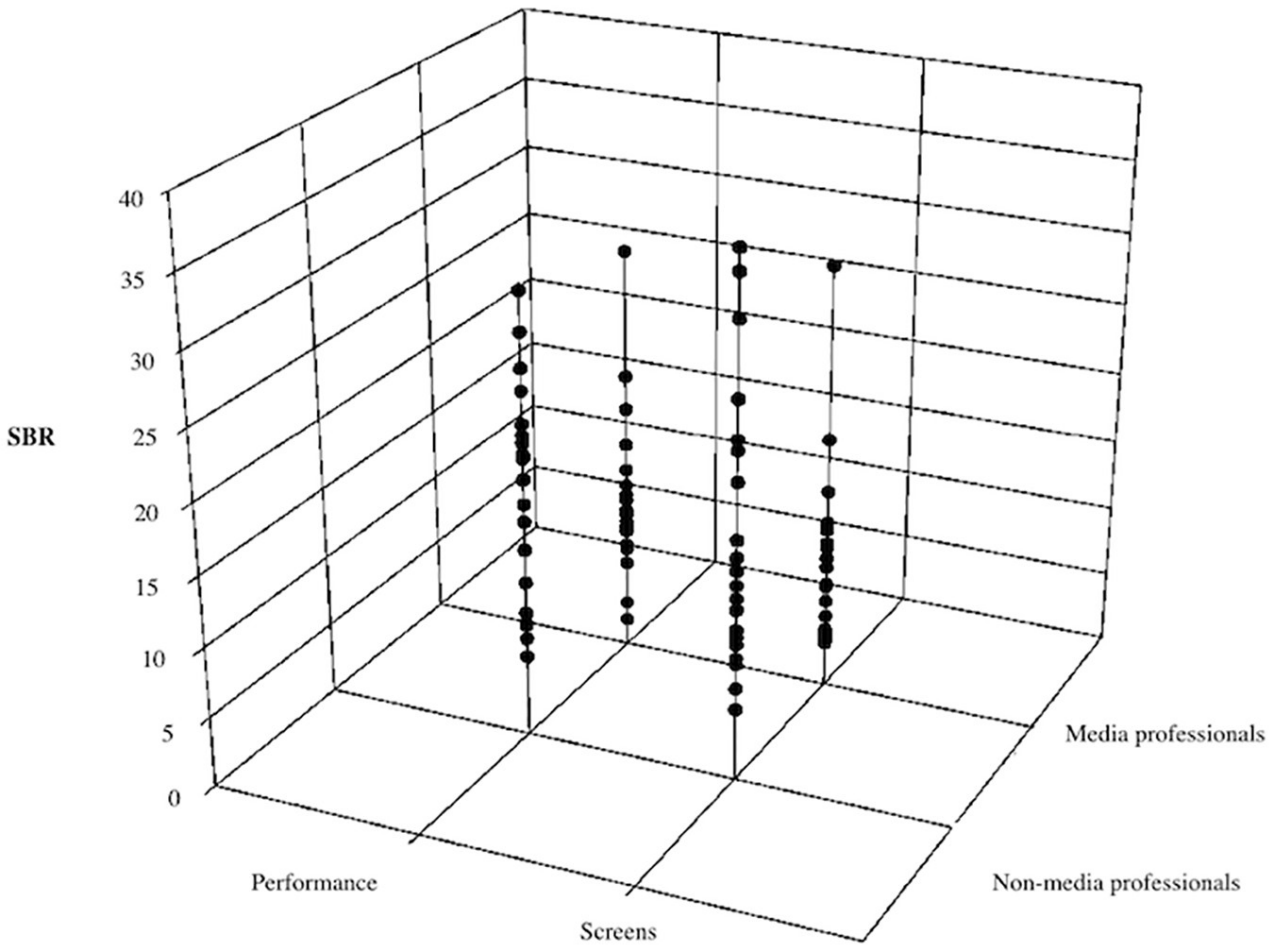
Screen and performance comparisons, in media and non-media professionals. 3D category scatter of SBR for the two factors analyzed: type of subject (media and non-media professionals), and type of stimulus (performance and screens). We can observe the trend of lower SBR in media professionals compared with non-media professionals; and the trend of an increase of SBR in performed play compared with screened stimuli.

The mean of the SBR in the totality of the subjects (N = 40) during the course of this experiment was 13.92 min^-1^ (*SD* = ± 8.342). In particular, the screened stimuli evoked an average of 13.208 eyeblinks min^-1^ (*SD* = ± 8.897), while the performed play evoked an average of 14.632 eyeblinks min^-1^ (*SD* = ± 7.794). There were big differences between groups: the screened stimuli evoked an average of 9.274 min^-1^ (*SD* = ± 6.453) for SBR in media-professionals (N = 20) and an average of 17.142 min^-1^ (*SD* = ± 9.395) for SBR in non-media professionals (N = 20), while the performed play evoked an average of 11.056 min^-1^ (*SD* = ± 6.042) for SBR in media professionals and 18.207 min^-1^ (*SD* = ± 7.828) in non-media professionals (Table 1, Fig 2). These results reveal a lower eyeblink rate when watching narrative scenes on screens compared with seeing them in a theatrical context. In addition, there seems to be a significantly lower rate of SBR in media professionals compared with non-media professionals in both situations: watching screens and looking at reality. Standard deviations also suggested a greater homogeneity in media professionals than in non-media professionals. We analyzed all these results to look for statistically significant differences.

**Table 1:** Spontaneous blink rate per minute.

Stimulus	Subjects	SBR min^-1^	S.D.
Screen	All subjects	13.208	8.897
Screen	Media professionals	9.274	6.453
Screen	Non-media professionals	17.142	9.395
Performance	All subjects	14.632	7.794
Performance	Media professionals	11.056	6.042
Performance	Non-media professionals	18.207	7.828

Descriptive results of blink rates per minute in screens (having the three editing styles) and in the performed play, in all subjects and by groups: media and non-media professionals.

The statistical analysis between looking at reality and watching screens showed interesting results. We compared SBRs obtained in subjects seeing a live play with those when watching the same narrative on screens. For that, we worked with the three most common video editing styles (sequence shot, Hollywood style, and MTV style). We also made the comparison between media and non-media professionals. We obtained significant differences in SBR between screens and reality: *F*_(1,38)_ = 5.384, *p* = 0.026 (two-way repeated-measures analysis of variance, ANOVA, one-factor repetition). We also obtained significant differences related to media professionalization: *F*_(1,38)_ = 10.607, *p* = 0.002 (two-way repeated-measures analysis of variance, ANOVA, one-factor repetition). No significant differences were obtained by crossing the two factors (*p*>0.05). We performed post hoc pairwise multiple comparison procedures following the Holm-Sidak method. We also observed that the type of stimulus (performed live play or on-screen movie), independently of the professionalization of the subject, affected SBR, *p*<0.05. Analyzing by groups, we found that media professionals’ SBR was affected by the type of stimulus, *p*<0.05. However, in the group of non-media professionals, the type of stimulus was not relevant, *p*>0.05. Their SBR was not affected whether looking at a live play or watching screens. We also found that media professionalization, irrespective of the type of stimulus, was a significant factor, *p*<0.001, for SBR. Finally, when comparing media professionalization within screens and within reality, in both cases we obtained highly significant statistical differences, *p*<0.01.

Thus, and according to our results, the type of stimulus (live performance or screened movie) in which a narrative is displayed affects the SBR of viewers. However, our results suggest that this is not so for non-media professionals, who seem to perceive live performances and screened movies similarly. The fact that media professionals show significant differences in their SBR in these two conditions may be connected to the higher level of attention that they are used to paying to screened stimuli. This would be coherent with the “dry eyes” problem in people watching screens with a high level of concentration sustained over time [15, 40–42]. Thus, these results suggest professionalization differences in visual perception in media professionals.

We also show here big differences in SBR between media and non-media professionals. Media professionals showed a significantly lower SBR watching screens (which seems logical as, in their everyday work, media professionals spend so many hours in front of audio-visual media making decisions related to edition on screens). Much more surprisingly, media professionals also showed a significantly lower SBR when seeing plays performed in the real world. This would be consistent with the idea that media professionalization affects visual perception.

## Discussion

We hypothesized that we would observe SBR differences between looking at the same narrative in reality and on-screen. We also hypothesized that we would find SBR differences in media professionals compared with non-media professionals. Interestingly, while our first hypothesis was partly supported, the second hypothesis was strongly supported. We obtained significant differences in SBRs between looking at reality vs. watching screens. However, in our analysis by groups, the results were not significant for the group of non-media professionals. It can be reasonably accepted that the visual behavior of non-media professionals is not different depending on the type of stimulus in which the narrative runs. They would pay the same attention to any stimulus, regardless of the format. We suggest that this result may be linked to the short duration of each stimulus, and we think that longer stimuli might have evoked differences between watching screens and looking at reality in non-media observers too. On the other hand, according to our results, we can assume that media professionals show a different SBR when watching a movie on a screen and when seeing the same story in a live performance. For media professionals, paying attention to screens is part of their job. Although we did not apply a specific protocol to determine subjects’ attention, the relationship between SBR and attention has been proven before [14]. Maybe, for that reason, whenever media professionals were facing screened stimuli their SBR dropped. This could suggest that media workers show a higher attentional level to decode the type of stimulus they are looking at.

As a second output of the present study, it can also be proposed that the viewer’s SBR varies depending on media professionalization. Earlier investigations had shown the relationship between professionalization and audio-visual behavior or brain structural changes [31–34]. Here, we found visual perception differences related to media professionalization. Our results showed not only that media professionals and non-media professionals behave differently facing screened stimuli, but that this difference is not restricted to visualization on screens. According to our investigation, media professionals present a lower SBR both when watching audio-visual works on screens and when seeing live plays performed in the real world. However, media workers visually distinguish between these two situations, and their SBR is, as mentioned above, different in each. According to Zheng et al. [43], more attention decreases eyeblinks and increases the level of mental workload. Thus, media professionals seem to present a higher level of attention to both screens and the real world than do non-professional observers. Our results also indicate that audio-visual works receive more attention than live performances. We suggest that advertising and marketing strategies could take this into account when forming communication strategies.

We know that today many non-media professionals also spend many hours a day making decisions related to on-screen content, as is the case of gamers. For that reason, and following this criterion, we wondered whether new experiments with gamers and media professionals would result in the same differences as found here. Our most surprising result is that the watching of screens steadily over time and making concomitant decisions with a high level of attention, as media professionals do, decreases SBR not only in media contexts, but also looking at live events. Taking into account today’s increased use of screens, we understand that it is important to keep on doing research following this line. These results invite us to continue searching for visual perception differences in media professionalization.

## Supporting information

S1 Dataset Spontaneous blink rate in screened movie and in live performance. (XLSX)

S1 Video Stimuli presentation. (MP4)
